# Human Tissue Processing and Transplantation in MESOT States: How to Promote?

**Published:** 2016-05-01

**Authors:** A. Heidary Rouchi, M. Mahdavi-Mazdeh

**Affiliations:** Iranian Tissue Bank and Research Center, Tehran University of Medical Sciences, Tehran, Iran

**Keywords:** Tissues, Human, Organ transplantation, Tissue and Organ Procurement, Tissue banks

## Abstract

Middle East, with more than 650 million inhabitants, has one the lowest mean donation rate in the world in the context of organ procurement from deceased donors with <2 per million population as actual deceased organ donation rate in 2014. Tissue processing and transplantation in this region are also restricted to a few countries among those representing Middle East Society for Organ Transplantation. Aiming to promote human tissue processing and transplantation in this region, as a life-enhancing therapeutic modality, we have to share our know-how and facilities in order so that the patients in different countries gain almost equal benefit of this vital procedure. To take different infrastructure of Middle Eastern countries into consideration and their plans to extend their activities, this intention will be come to the reality and materialized by primarily sharing the processed tissues among member states in a centralized manner and to offer processing services and banking to locally retrieved tissues.

## INTRODUCTION

The Middle East Society for Organ Transplantation (MESOT) is being represented by more than 20 countries with more than 650 million inhabitants. The history and the extent of activities in the field of organ and tissue procurement and transplantation in these countries follow a heterogeneous pattern. According to international registries, nine out of these countries (*i.e.*, Iran, Kuwait, Turkey, Saudi Arabia, Lebanon, Tunisia, Qatar, Morocco, and Algeria) were active in the context of organ procurement from deceased donors with less than two per million population (PMP) as mean donation rate in 2014, compared to global rates ranged from 0.02 in Algeria to 35.9 in Spain. Most of the above-mentioned countries are active in the field of tissue procurement and processing from deceased donors that in most cases, are restricted to merely a few types of tissues [1-5].

Religious, cultural and legislative barriers, not optimal infrastructure, and lack of resources are the main reasons to make a country deprived from this life-saving and life-enhancing therapeutic modality. Contrary to ethnic diversity in MESOT covering region, Islam is the prevaling religion and there is no such a prominent religious variety that requires different approaches. Organ and tissue donation and transplantation are widely praised in all sects of Islam, with rare exceptions, though [6-9]. Yet, we face a broad spectrum in MESOT states in viewpoint of legislative and socioeconomic status; consequently, to formulate a unique approach and provide the MESOT countries with a single strategy to promote these activities cannot help us to achieve our long-term goals [10]. 

The first step to highlight the importance of tissue procurement was taken in the 14^th^ MESOT congress in September 2014, Istanbul, Turkey. To set up a designed and specific committee for tissue and cell transplantation was in line with this inevitable significance.

To consider the perception of “death” among general population convinces us that people face the death with cardio-respiratory manifestations much more believable compared with death with brain criteria. This fact makes establishment a tissue procurement program in a region easier that in turn can pave the way to set up a more extended program to work on organ procurement from deceased donors.

**STRATEGIC FOCUS**

This plan aims mainly to promote the activity in the field of human tissue processing and transplantation in MESOT countries, which is unintentionally neglected for many years for potentially solvable barriers. Different countries in our region have different values and standards and hence various approaches should be undertaken in parallel with concentrating on common concerns. To design and implement a specific plan for every country is the key to fulfill a country’s objectives. Countries in the region have different infrastructures, and health priorities, so the priority of this plan is to primarily start working with countries with already set up system on organ and tissue procurement from deceased donors. 

In this regard, the regional countries can be categorized into four groups:

Countries with running program on organ and tissue procurement from deceased donors, even with experience on few types of tissues.

Countries with active program on organ procurement and transplantation from deceased donors, but no activities in the field of tissue procurement.

Countries with no operational system on organ or tissue procurement from deceased donors due to one or more religious, cultural, legislative or funding barriers but with stable political situation.

Countries with the 3^rd^ group features but with presently internal conflicts.

Our plan diverts all these classified countries with different interventions. To grossly have a look at this plan, the designed programs can be chronologically listed:

Short-term Program

This program is going to be run throughout 2016. The main activities in this phase are:

To set up MESOT Committee for Tissue Procurement and Transplantation.

To finalize the bylaws, strategic and action plans.

To make regular connection and conduct meetings with local tissue procurement committees in all member states.

To make a centralized profile of every country in terms of their situation and plans in the field of given subject.

Mid-term Program

This program also begins from 2016 and is scheduled to be terminated at the end of this year. This program is mainly applicable to the countries with already established system on organ and/or tissue procurement from deceased donors. To upgrade the currently running systems and to create fundamentals to work on the subject of tissue procurement and transplantation is the main goal of this program.

Long-term Program

This program is to settle and stabilize all the above-mentioned activities and promote other countries to join the active countries. It is presumed to be lasted until the end of 2018.

**MEMBER STATES**

Target countries for this plan comprehend but not limited to following alphabetically sorted countries: Algeria, Bahrain, Egypt, Iran, Iraq, Jordan, Saudi Arabia, Kuwait, Lebanon, Libya, Morocco, Oman, Pakistan, Palestine, Qatar, Sudan, Syria, Tunisia, Turkey, United Arab Emirates, and Yemen.

**MESOT COMMITTEE FOR HUMAN TISSUE AND CELL TRANSPLANTATION**

This committee will officially start its activity from the beginning of 2016. All MESOT member states can have at least one member (maximum of 2) in this committee. The secretariat will be based in Iranian Tissue Bank (ITB), Tehran, Iran.

The tasks and general policies will be cleared in a regular basis and based on general consensus. All activities in member countries concerning tissue procurement will be reported to and archived in the committee secretariat for further surveillances and interventions.

To finalize the bylaws, strategic plan and action plan is among committee’s tasks with high priority. There will be Executive, Ethical, and Technical subcommittees. To guarantee the efficacy of all interventions in each country, all activities should be first approved by the committee.

**IMPLEMENTATION OF DIFFERENT POSSIBLE INTERVENTIONS**

Different scenarios for various interventions are hereunder short-listed. These approaches are subject to modifications based on every country’s current situation; these are also flexible in the course of implementation for any unforeseen reactions:

Countries with no barriers and enough funding can take advantage of knowledge, expertise, and technology transfer from currently active countries in the region. An inter-regionally group with different nationalities will be teamed up by the committee, accordingly. Training programs and running workshops are also the tasks of this team, once required.

Countries with no infrastructure can receive the processed tissues for transplantation from other member states with ongoing program on tissue procurement. The allocation by itself is done virtually by the committee following the finalization of allocation criteria. The reimbursement rates (on tissue basis) will be determined by the committee and cover just processing and shipment costs.

Countries with facilities for tissue retrieval but with no tissue bank can ship the locally retrieved tissue to countries with tissue processing and banking possibilities to benefit from their expertise and infrastructure. The senders receive the processed tissues and reimburse only the processing and shipment costs.

**SURVEILLANCE AND AUDIT (INTER-REGIONAL AND EXTERNAL)**

All activities, interventions and co-operations are supervised by the committee in a regular basis. Documentation and archive are with high level of importance for further follow-up. Techniques to assess donor eligibility will be agreed and finalized in technical subcommittee. All documentations are subject to control based on released guidelines/protocols. Inter-regional and time to time external audit plans are among the vital procedures that guarantee the safety and efficacy of products.

**OTHER CONSIDERATIONS**

In order to make a legal framework, main correspondences between member states (once required) will be changed through respective Ministries of Health and Embassies. Every country’s committee for tissue procurement and transplantation is in charge of making all required local connections to obtain all necessary authorizations to start activity in the field of tissue procurement and transplantation.

## CONCLUSION

For a patient with damaged or non-functioning tissue, allograft is the replacement of choice. The highest biocompatibility and near-native performance make human tissues as the best option. Nevertheless, for the most countries, given more emphasis to the Middle East, tissue processing and transplantation are not properly established practices. To share the expertise and facilities in a well-designed collaborative and regional network make this life-improving therapeutic modality accessible for all patients. 

## Notes

**FINANCIAL SUPPORT:** None.
